# Cost-effectiveness of anesthesia maintained with sevoflurane or propofol with and without additional monitoring: a prospective, randomized controlled trial

**DOI:** 10.1186/s12871-018-0563-z

**Published:** 2018-07-28

**Authors:** Timea Bocskai, Csaba Loibl, Zoltan Vamos, Gabor Woth, Tihamer Molnar, Lajos Bogar, Laszlo Lujber

**Affiliations:** 1 Department of Anaesthesiology and Intensive Therapy, University of Pécs, Medical School, Ifjúság Str 13, Pécs, 7624 Hungary; 2 Department of Otorhinolaryngology, University of Pécs, Medical School, Pécs, Hungary

**Keywords:** Anesthesia, Patient safety, Cost, Bispectral index, Train-of-four

## Abstract

**Background:**

We compared cost-effectiveness of anesthesia maintained with sevoflurane or propofol with and without additional monitoring, in the clinical setting of ear-nose-throat surgery.

**Methods:**

One hundred twenty adult patients were randomized to four groups. In groups SEVO and SEVO+ anesthesia was maintained with sevoflurane, in group SEVO+ with additional bispectral index (BIS) and train-of-four (TOF) monitoring. In groups PROP and PROP+ anesthesia was maintained with propofol, in group PROP+ with additional BIS and TOF monitoring.

**Results:**

Total cost of anesthesia per hour was greater in group SEVO+ compared to SEVO [€ 19.95(8.53) vs. 12.15(5.32), *p* <  0.001], and in group PROP+ compared to PROP (€ 22.11(8.08) vs. 13.23(4.23), *p* <  0.001]. Time to extubation was shorter in group SEVO+ compared to SEVO [11.1(4.7) vs. 14.5(3.9) min, *p* = 0.002], and in PROP+ compared to PROP [12.6(5.4) vs. 15.2(4.7) min, *p* <  0.001]. Postoperatively, arterial blood pressure returned to its initial values sooner in groups SEVO+ and PROP+.

**Conclusions:**

Our study demonstrated that the use of BIS and TOF monitoring decreased the total cost of anesthesia drugs and hastened postoperative recovery. However, in our circumstances, these were associated with higher disposables costs. Detailed cost analysis and further investigations are needed to identify patient populations who would benefit most from additional monitoring.

**Trial registration:**

ClinicalTrials.gov, NCT02920749. Retrospectively registered (date of registration September 2016).

## Background

Inadequate depth of anaesthesia carries significant morbidity. Greater than required depth of anaesthesia may be associated with increased risk of complications and higher cost of anaesthesia. This may also prolong recovery times and potentially increase health-care costs [1, 2]. Inadequately light anaesthesia may increase the risk of intraoperative awareness [3]. Similarly, incomplete reversal of neuromuscular blockade results in residual neuromuscular weakness with all its undesirable consequences [4, 5].

Monitoring techniques may potentially reduce complications of anaesthesia in the perioperative period [6]. Only few studies published on simultaneous monitoring of depth of anaesthesia with bispectral index (BIS) and of neuromuscular blockade with train-of-four (TOF) [7].

Cost-effectiveness requires the most effective distribution and use of available resources in order to reach maximal health gain. The cost of a certain activity, treatment, working process is made up of fixed and variable parts [8]. Cost analyses must balance the costs of various agents, pharmacodynamic advantages of anesthetics (e.g. sevoflurane, propofol), perioperative complications, and patient monitoring techniques [9, 10]. The majority of studies on cost-effectiveness focus only on the cost of maintaining anesthesia. Others examine cost-effectiveness taking into account only additional costs and cost-cutting factors [11–13]. With total intravenous anesthesia techniques the need for disposables (syringe, extension tube for infusion set, valve) increases total cost [14, 15]. Some studies compared the cost of anesthesia with BIS vs. hemodynamic monitoring [16–18].

The aim of our study was to compare drug dosage and total cost (direct plus additional costs) of anesthesia maintained with sevoflurane or propofol with or without the combination of BIS and TOF monitoring respectively. Our hypothesis was that the total cost of sevoflurane or propofol anesthesia combined with both BIS and TOF is less than without BIS and TOF.

## Methods

### Patient selection

Approval for this prospective, randomized study was obtained from the Regional Research Ethics Committee of the Medical Center, University of Pécs and it was retrospectively registered in the ClinicalTrials.gov (NCT02920749). All patients had been informed about the investigation and had signed the Informed Consent before anesthesia. The study took place at the Department of Otorhinolaryngology, University of Pécs, Medical School, Hungary. Between September 2014 and October 2016 American Society of Anesthesiologists (ASA) I or II patients aged 18–65 years, scheduled for elective Ear-nose-throat (ENT) surgery and anesthesia with controlled hypotension (tympanoplasty, parotidectomy or septoplasty), were recruited. Exclusion criteria were bronchial asthma, chronic obstructive pulmonary disease, epilepsy, psychiatric illness, cerebrovascular or congenital neuromuscular disease. Patients were randomized to one of four anesthetic treatment groups. The allocation sequence was concealed from researchers (TB, CL and ZV) enrolling and assessing participants in sequentially numbered, sealed and stapled envelopes.

### Study protocol

Midazolam 7.5 mg was administered orally 1.5 h before induction of anesthesia. Ringer lactate was used as background infusion at the rate of 5 ml kg^− 1^ h^− 1^ throughout anesthesia. Electrocardiography (ECG), invasive mean arterial blood pressure (MAP), heart rate, peripheral capillary oxygen saturation (SpO_2_), and end-tidal carbon dioxide were continuously monitored and recorded at 5 min intervals intraoperatively. Anesthesia of groups SEVO and PROP did not entail BIS or TOF monitoring where as that of groups SEVO+ and PROP+ was guided by BIS (BIS® Quatro Brain Monitoring Sensor, Aspect Medical Systems, Inc., Norwood, MA, USA) and TOF monitoring (Infinity®, Trident® NMT SmartPod®, Dräger Medical Systems, Inc., Danvers, MA, USA). Following four stimuli delivered every 0.5 s with 2 Hz frequency, contractions of the adductor pollicis muscle were measured using acceleromyography. BIS and TOF values were recorded at 5 min intervals.

Anesthesia was induced with fentanyl, propofol and atracurium in all groups. After five minutes of preoxygenisation with 100% oxygen, an initial dose of fentanyl 1 μg kg^− 1^ was administered intravenously (IV). In groups SEVO and PROP, anesthesia was induced with IV propofol titrated to loss of eyelash-reflex. In groups SEVO+ and PROP+, the induction agent propofol was titrated to a BIS value of 90 at the end of bolus injection. Due to the time delay between propofol bolus and endotracheal intubation BIS decreases further to the maintenance target range of 40–60 [19, 20]. An intubation dose of 0.5 mg kg^− 1^ IV atracurium was administered to all patients. In groups SEVO and PROP, tracheal intubation was carried out after 4 min. In groups SEVO+ and PROP+, tracheal intubation was attempted only when a BIS value less than 60 and a TOF count of zero were achieved.

In group SEVO, anesthesia was maintained with sevoflurane with a target of aged-adjusted minimal alveolar concentration (MAC) 1.0–1.5 in air and oxygen mixture with inspired oxygen fraction (FiO_2_) of 0.50. In group PROP, anesthesia was maintained with propofol (with Roberts “10–8-6” scheme) with the same FiO_2_ [21]. In groups SEVO+ and PROP+, sevoflurane or propofol dosage was set to maintain target BIS levels of 40 to 60. Fresh gas flow (FGF) was 1 L min^− 1^ during anesthesia in all groups.

In groups SEVO and PROP the repeat dose of atracurium 0.15 mg kg^− 1^ was given every 30 min during anesthesia. In groups SEVO+ and PROP+ the neuromuscular block level was maintained with a TOF monitor at the level of one or no response. Further 0.15 mg kg^− 1^ atracurium was administered as required.

Target mean arterial pressure (MAP) was set between 60 and 85 mmHg intraoperatively. If MAP increased by more than 20%, a fentanyl bolus of 50 μg was administered in all groups.

At the end of surgery sevoflurane and propofol were stopped 5–10 min before the completion of surgery in all groups. Sevoflurane was stopped but FGF was left on low level until the end of surgery (wound cover of surgical area). Neostigmine and atropine (2.5 and 1.0 mg) were administered as neuromuscular block reversal to all patients. In groups SEVO and PROP the total reversal amount was given and spontaneous ventilation was established prior to extubation. In groups SEVO+ and PROP+ the reversal mixture was incrementally given until a TOF ratio of > 0.9 was reached. Once extubated all patients received 35% O_2_ through a Venturi face mask and were transferred to the recovery room. Vital parameters were monitored and recorded every 15 min for two hours. Diclofenac (75 mg) and nalbuphine (5–10 mg) were administered as rescue analgesics.

Minor perioperative complications of general anesthesia (hypotension, postoperative nausea and vomiting (PONV), sore throat, headache, drowsiness, dizziness, cognitive dysfunction, memory loss, vision problems, shivering, and myalgia) were recorded.

Cost analysis included all drugs and disposables (e.g. syringes, needles, infusion lines, three-way stop-cocks, electrodes, suction catheters, endotracheal tubes, BIS sensors). We used injection vials for multiple patients but disposables were changed for each patient.

The cost of sevoflurane used was calculated with the following formula, adapted from previous studies [22]:

$$ \mathrm{Cost}\left(\text{\EUR} \right)=\frac{\mathrm{FGF}\left(\mathrm{L}\ {\min}^{\hbox{-} 1}\right)\times \mathrm{Sevoflurane}\ \mathrm{Vol}\%\times \mathrm{Duration}\left(\min \right)\times \mathrm{Cost}\ \mathrm{of}\ 1\ \mathrm{bottle}\left(\text{\EUR} \right)}{\mathrm{Liquid}\ \mathrm{to}\ {\mathrm{vapor}\ \mathrm{ratio}}^{\ast}\times \mathrm{Volume}\ \mathrm{of}\ \mathrm{a}\ \mathrm{bottle}{\left(\mathrm{L}\right)}^{{{}^{\ast}}^{\ast }}} $$

(* Liquid to vapor ratio for sevoflurane: 183 ml; ** Bottle volume of sevoflurane: 0.25 L)

Our estimates were based on the information obtained from the pharmacy about the costs in euros (€).

### Primary and secondary outcome

Our hypothesis was that total drug cost of sevoflurane and propofol anesthesia with BIS and TOF is lower compared to anesthesia without BIS and TOF. Our primary outcome was the total cost of anesthesia. Secondary outcomes were time spent in theatre and recovery room.

Time intervals in theatre and recovery room were defined as follows: length of anesthesia was the time from induction until extubation; length of surgery was the time from skin incision until wound cover of surgical area; time to extubation was the time between end of surgery and extubation; “time to MAP restoration” was the time from admission in recovery room until MAP returned to preoperative level (within 5% of initial value).

### Statistical analysis

Statistical analysis was carried out with SPSS version 21 for Windows (IBM Corporation) software. All data are expressed as mean ± standard deviation (SD). Nonparametric test, Kruskal-Wallis (k-sample) with pairwise comparison was performed to assess the differences between groups. A *p* value of <  0.05 was considered statistically significant. Based on sample size estimation (type I α = 5% and with type II (power) of 90%), 27 patients are needed to detect 15% reduction in total cost of anesthesia.

## Results

Patients’ demographic characteristics are presented in Table 1. Surgical characteristics were similar and comparable in all groups. In group SEVO were 17 tympanoplasty, 3 parotidectomy and 2 septoplasty. In group SEVO+ were 22 tympanoplasty, 4 parotidectomy and 2 septoplasty. In group PROP were 22 tympanoplasty, 4 parotidectomy and 4 septoplasty. In group PROP+ were 19 tympanoplasty, 6 parotidectomy and 4 septoplasty.

**Table 1:** Patient characteristics

	SEVO (*n* = 30)	SEVO+ (*n* = 30)	*p* value	PROP (*n* = 30)	PROP+ (*n* = 29)	*p* value
Sex (female/male)	14/16	16/14	1.000	19/11	16/13	1.000
Age (years)	48 ± 16	38 ± 15	0.112	42 ± 14	41 ± 13	1.000
Body weight (kg)	80 ± 16	77 ± 18	1.000	72 ± 15	74 ± 16	1.000
Height (cm)	175 ± 12	172 ± 17	1.000	173 ± 10	172 ± 15	1.000
ASA I/II	14/16	17/13	1.000	18/12	17/12	1.000

Values are expressed as mean ± standard deviation; *SEVO* Sevoflurane anesthesia without additional monitoring; *SEVO+* Sevoflurane anesthesia with BIS and TOF monitoring; *PROP* Propofol anesthesia without additional monitoring; *PROP+* Propofol anesthesia with BIS and TOF monitoring; *ASA* American Society of Anesthesiologists physical status; gender and ASA grade are the number of patients

**Fig. 1:**
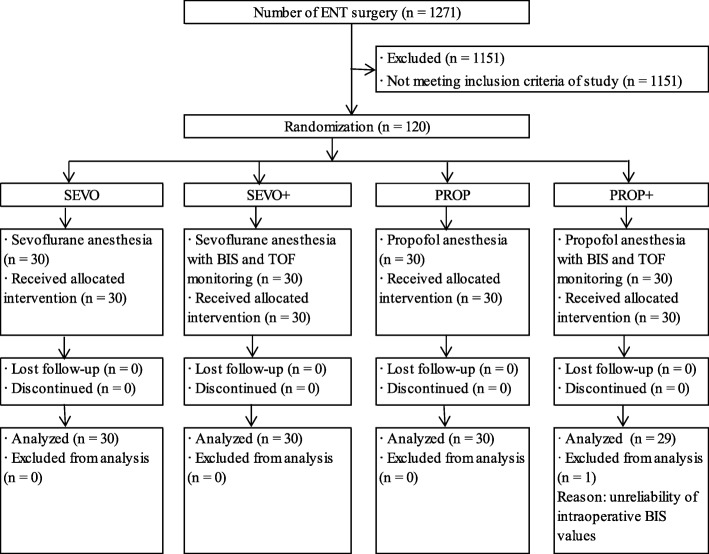
The flow diagram of the study

The CONSORT Flow Diagram is reported in Fig. 1. One patient was excluded from analysis because the unreliability of intraoperative BIS values.

The intraoperative target range of controlled hypotension was unequivocally achieved in all groups. Intraoperative MAP was lower in group SEVO compared to PROP [71(10) vs. 78(9) mmHg, *p =* 0.029] and group SEVO+ compared to PROP+ [70(8) vs. 84(11) mmHg, *p* <  0.001]. Intraoperative heart rate was similar in group SEVO compared to SEVO+ and in group PROP compared to PROP+. Intraoperative heart rate was lower in group SEVO compared to PROP [64(15) vs. 73(10) bpm, *p*  = 0.019]. Intraoperative BIS and TOF values were similar in both groups with BIS and TOF monitoring (groups SEVO+ and PROP+) (Table 2).

**Table 2:** Intraoperative MAP, heart rate, BIS, and TOF parameters

Intraoperative parameters	SEVO (*n* = 30)	SEVO+ (*n* = 30)	*p* value	PROP (*n* = 30)	PROP+ (*n* = 29)	*p* value
*MAP (mmHg)*	71 ± 10	70 ± 8	N.S.	78 ± 9#	84 ± 11##	N.S.
*Heart rate (bpm)*	64 ± 15	67 ± 11	N.S.	73 ± 10*	70 ± 7	N.S.
*BIS*						
Before anesthesia	–	97 ± 2		–	97 ± 2	N.S.
After propofol bolus at induction	–	87 ± 7		–	86 ± 7	N.S.
At intubation		51 ± 9		–	56 ± 3	N.S.
During intraoperative period	–	47 ± 5		–	49 ± 9	N.S.
*TOF*	–	2 ± 1		–	2 ± 1	N.S.

Values are expressed as mean ± standard deviation; *MAP* Mean arterial blood pressure; *BIS* Bispectral index; *TOF* Train-of-four; *SEVO* Sevoflurane anesthesia without additional monitoring; *SEVO*+ Sevoflurane anesthesia with BIS and TOF monitoring; *PROP* Propofol anesthesia without additional monitoring; *PROP*+ Propofol anesthesia with BIS and TOF monitoring; *: SEVO-PROP *p*= 0.019; #: SEVO-PROP *p*=0.029; ##: SEVO+-PROP+ *p*< 0.001; *N.S*. No significant different

*Doses of anesthetic drugs* varied amongst the groups. Induction dose of propofol was less in group SEVO+ compared to SEVO [166.4(35.2) vs. 196.3(46.9) mg, *p* <  0.001] and in group PROP+ compared to PROP [147.3(30.2) vs. 194.3(18.9) mg, *p* <  0.001]. It was however similar in groups SEVO and PROP, and in groups SEVO+ and PROP+. Propofol dose for induction was less in group SEVO+ compared to SEVO [2.1 (0.6) vs. 2.5 (0.7) mg kg^− 1^, *p* = 0.009] and in group PROP+ compared to PROP [2.1 (0.5) vs. 2.9 (0.6) mg kg^− 1^, *p* <  0.001]. Hourly maintenance doses of fentanyl and atracurium were similar in all groups (Table 3).

**Table 3:** Perioperative anesthetic drugs consumption

Drugs	SEVO (*n* = 30)	SEVO+ (*n* = 30)	*p* value	PROP (*n* = 30)	PROP+ (*n* = 29)	*p* value
Induction of anesthesia						
Fentanyl (μg)	98.3 ± 9.1	103.3 ± 18.3	1.000	99.1 ± 4.6	99.2 ± 10.3	1.000
Propofol (mg)	196.3 ± 46.9	166.4 ± 35.2	< 0.001	194.3 ± 18.9	147.3 ± 30.2	< 0.001
Propofol (mg kg^− 1^)	2.5 ± 0.7	2.1 ± 0.6	0.009	2.9 ± 0.6	2.1 ± 0.5	< 0.001
Atracurium (mg)	38.3 ± 4.2	37.3 ± 6.7	1.000	36.0 ± 5.7	37.7 ± 7.0	1.000
Conduct of anesthesia						
Fentanyl (μg/hour)	54.6 ± 26.9	59.8 ± 38.9	1.000	89.8 ± 42.8	94.7 ± 37.0	0.996
Sevoflurane (ml/hour)	14.4 ± 2.8	11.8 ± 12.4	0.864	–	–	
Propofol (mg/hour)	–	–		1185.5 ± 320.9	1082.1 ± 297.9	1.000
Atracurium (mg/hour)	8.3 ± 3.7	7.4 ± 4.7	1.000	8.5 ± 4.7	8.9 ± 6.1	1.000

Values are expressed as mean ± standard deviation; *SEVO* Sevoflurane anesthesia without additional monitoring; *SEVO*+ Sevoflurane anesthesia with BIS and TOF monitoring; *PROP* Propofol anesthesia without additional monitoring; *PROP*+ Propofol anesthesia with BIS and TOF monitoring

*The cost* of propofol as induction agent was 19.0% less in group SEVO+ compared to SEVO [€ 0.94(0.19) vs. 1.16(0.16), *p* = 0.016], and 28.9% less in group PROP+ compared to PROP [€ 0.81(0.20) vs. 1.14(0.08), *p* <  0.001]. Hourly costs of fentanyl, sevoflurane and atracurium were similar in groups SEVO and SEVO+. Hourly costs of fentanyl, propofol and atracurium were similar in groups PROP and PROP+, respectively (Table 4). However, the hourly cost of fentanyl was less in group SEVO compared to PROP [€ 0.12(0.08) vs. 0.18(0.08), *p* <  0.001], and in group SEVO+ compared to PROP+ [€ 0.12(0.09) vs. 0.20(0.07), *p* <  0.001) (Table 5)*.*

**Table 4:** Cost elements of anesthesia I – group SEVO compared to SEVO+ and group PROP compared to PROP+

Costs	SEVO (*n* = 30)	SEVO+ (*n* = 30)	*p* value	PROP (*n* = 30)	PROP+ (*n* = 29)	*p* value
and in group PROP(€)						
Fentanyl	0.19 ± 0.03	0.20 ± 0.03	1.000	0.19 ± 0.02	0.20 ± 0.02	1.000
Propofol	1.16 ± 0.16	0.94 ± 0.19	0.016	1.14 ± 0.08	0.81 ± 0.20	< 0.001
Atracurium	2.44 ± 0.27	2.37 ± 0.42	1.000	2.22 ± 0.55	2.30 ± 0.36	1.000
Conduct of anesthesia (€/hour)						
Fentanyl	0.12 ± 0.08	0.12 ± 0.09	1.000	0.18 ± 0.08	0.20 ± 0.07	0.996
Sevoflurane	4.62 ± 2.03	4.12 ± 1.61	0.864	–	–	
Propofol	–	–		3.39 ± 0.91	3.08 ± 0.85	1.000
Atracurium	0.58 ± 0.40	0.52 ± 0.42	0.292	0.67 ± 0.55	0.56 ± 0.39	1.000
Other drugs (€/hour)	1.51 ± 2.14	1.22 ± 1.99	1.000	1.83 ± 1.15	1.60 ± 2.01	0.572
Summary of costs						
Total drug (€/hour)	8.84 ± 4.11	7.86 ± 3.54	0.002	8.33 ± 3.02	7.52 ± 2.49	< 0.001
Total disposables (€)	6.49 ± 0.11	23.25 ± 0.12	< 0.001	8.09 ± 0.07	24.76 ± 0.19	< 0.001
Total cost of anesthesia (€/hour)	12.15 ± 5.32	19.95 ± 8.53	< 0.001	13.23 ± 4.23	22.11 ± 8.08	< 0.001

Values were expressed as mean ± standard deviation; *SEVO* Sevoflurane anesthesia without additional monitoring; *SEVO*+ Sevoflurane anesthesia with BIS and TOF monitoring; *PROP* Propofol anesthesia without additional monitoring; *PROP*+ Propofol anesthesia with BIS and TOF monitoring; *Other drugs* antidotes (neostigmine and atropine mixture, nalbuphine, and flumazenil) and postoperative pain management

**Table 5:** Cost elements of anesthesia II – group SEVO compared to PROP and group SEVO+ compared to PROP+

Costs	SEVO (*n* = 30)	PROP (*n* = 30)	*p* value	SEVO+ (*n* = 30)	PROP+ (*n* = 29)	*p* value
Induction of anesthesia (€)						
Fentanyl	0.19 ± 0.03	0.19 ± 0.02	1.000	0.20 ± 0.03	0.20 ± 0.02	1.000
Propofol	1.16 ± 0.16	1.14 ± 0.08	0.996	0.94 ± 0.19	0.81 ± 0.20	0.205
Atracurium	2.44 ± 0.27	2.22 ± 0.55	0.253	2.37 ± 0.42	2.30 ± 0.36	1.000
Conduct of anesthesia (€/hour)						
Fentanyl	0.12 ± 0.08	0.18 ± 0.08	< 0.001	0.12 ± 0.09	0.20 ± 0.07	< 0.001
Atracurium	0.58 ± 0.40	0.67 ± 0.55	1.000	0.52 ± 0.42	0.56 ± 0.39	1.000
Hypnotic agent	4.62 ± 2.03	4.12 ± 1.61	< 0.001	3.39 ± 0.91	3.08 ± 0.85	< 0.001
Other drugs (€/hour)	1.51 ± 2.14	1.83 ± 1.15	0.958	1.22 ± 1.99	1.60 ± 2.01	1.000
Summary of costs						
Total drug (€/hour)	8.84 ± 4.11	8.33 ± 3.02	< 0.001	7.86 ± 3.54	7.52 ± 2.49	< 0.001
Total disposables (€)	6.49 ± 0.11	8.09 ± 0.07	< 0.001	23.25 ± 0.12	24.76 ± 0.19	< 0.001
Total cost of anesthesia (€/hour)	12.15 ± 5.32	13.23 ± 4.23	< 0.001	19.95 ± 8.53	22.11 ± 8.08	< 0.001

Values are expressed as mean ± standard deviation; *SEVO* Sevoflurane anesthesia without additional monitoring; *SEVO*+ Sevoflurane anesthesia with BIS and TOF monitoring; *PROP* Propofol anesthesia without additional monitoring; *PROP*+ Propofol anesthesia with BIS and TOF monitoring; Hypnotic agent in groups SEVO and SEVO+ was sevoflurane; Hypnotic agent in groups PROP and PROP+ was propofol; *Other drugs* Antidotes (neostigmine and atropine mixture, nalbuphine, and flumazenil) and postoperative pain management

Total hourly drug cost was 11.1% less in group SEVO+ compared to SEVO [€ 7.86(3.54) vs. 8.84(4.11), *p* = 0.002], and 9.7% less in group PROP+ compared to PROP [€ 7.52(2.49) vs. 8.33(3.02), *p* <  0.001]. Total cost of anesthesia disposables was greater in group SEVO+ compared to SEVO [€ 23.25(0.12) vs. 6.49(0.11), *p* <  0.001], and in group PROP+ compared to PROP [€ 24.76(0.19) vs. 8.09(0.07), *p* <  0.001]. Total hourly cost of anesthesia was 64.2% greater in group SEVO+ compared to SEVO [€ 19.95(8.53) vs. 12.15(5.32), *p* <  0.001], and 67.1% greater in group PROP+ compared to PROP [€ 22.11(8.08) vs. 13.23(4.23), *p* <  0.001] (Table 4).

Costs of reversal and postoperative pain relief (cost of other drugs) were similar in all groups (Table 4).

Hourly cost of hypnotic agent was greater in group SEVO compared to PROP [€ 4.62(2.03) vs. 4.12(1.61), *p* <  0.001], and in group SEVO+ compared to PROP+ [€ 3.39(0.91) vs. 3.08(0.85), *p* <  0.001]. Total hourly cost of anesthesia drugs was greater in group SEVO compared to PROP [€ 8.84(4.11) vs. 8.33(3.02), *p* <  0.001], and in group SEVO+ compared to PROP+ [€ 7.86(3.54) vs. 7.52(2.49), *p* <  0.001], respectively. Total cost of anesthesia disposables was less in group SEVO compared to PROP [€ 6.49(0.11) vs. 8.09(0.07), *p* <  0.001], and in group SEVO+ compared to PROP+ [€ 23.25(0.12) vs. 24.76(0.19), *p* <  0.001]. Total hourly cost of anesthesia was less in group SEVO compared to PROP [€ 12.15(5.32) vs. 13.23(4.23), *p* <  0.001], and in group SEVO+ compared to PROP+ [€ 19.95(8.53) vs. 22.11(8.08), *p* <  0.001], respectively (Table 5).

In the early postoperative period the incidence of minor complications was similar in all groups. Time to extubation and time to MAP restoration were similar in groups SEVO vs. PROP and SEVO+ vs. PROP+. Time to extubation was shorter in group SEVO+ compared to SEVO [11.1(4.7) vs. 14.5(3.9) min, *p* = 0.002], and in group PROP+ compared to PROP [12.6(5.4) vs. 15.2(4.7) min, *p* <  0.001], respectively. Time to MAP restoration was similar in group SEVO+ compared to SEVO and in group PROP+ compared to PROP. Length of patient stay in recovery room was similar in all groups (Table 6).

**Table 6:** Data and recovery profiles in perioperative period of anesthesia

	SEVO (*n* = 30)	SEVO+ (*n* = 30)	*p* value	PROP (*n* = 30)	PROP+ (*n* = 29)	*p* value
Length of anesthesia (min)	140.7 ± 55.5	137.0 ± 53.0	0.998	115.8 ± 53.4	124.4 ± 54.9	0.997
Length of surgery (min)	99.7 ± 51.5	103.5 ± 49.0	1.000	81.2 ± 46.2	89.2 ± 51.0	1.000
Time to extubation (min)	14.5 ± 3.9	11.1 ± 4.7	0.002	15.2 ± 4.7	12.6 ± 5.4	< 0.001
PONV	1(3.3%)	2(6.7%)	0.990	1(3.3%)	1(3.5%)	1.000
Other minor complications	13(43.3%)	13(43.3%)	1.000	3(10.0%)	7(24.1%)	1.000
Time to MAP restoration (min)	15.2 ± 7.6	12.3 ± 6.0	0.517	17.2 ± 9.5	11.1 ± 10.0	0.076
Length of patient stay in recovery room (min)	69.5 ± 37.9	63.8 ± 16.1	0.454	64.3 ± 29.7	62.8 ± 12.4	0.799

Values are expressed as mean ± standard deviation; *SEVO* Sevoflurane anesthesia without additional monitoring; *SEVO*+ Sevoflurane anesthesia with BIS and TOF monitoring; *PROP* Propofol anesthesia without additional monitoring; *PROP*+ Propofol anesthesia with BIS and TOF monitoring; *PONV* Postoperative nausea and vomiting; Other minor complications Sore throat, headache, drowsiness, dizziness, cognitive dysfunction, memory loss, vision problems, shivering, and myalgia; Other minor complications are the number of patients and incidence rates (%); MAP = mean arterial blood pressure

## Discussion

The findings of this study confirm the hypothesis that the total drug cost of sevoflurane and propofol anesthesia with BIS and TOF is lower than without BIS and TOF monitoring. Although BIS monitoring decreased the total cost of anesthesia drugs, it pushed up the total cost of anesthesia. Furthermore, this study demonstrated that using TOF monitoring did not influence the cost of atracurium during anesthesia.

Klopman et al. demonstrated that use of hypnotic anesthetic drugs, time to extubation, incidence of nausea and/or vomiting, and intraoperative awareness were decreased when BIS monitor was used [16]. Shepherd et al. demonstrated that the general anesthetic consumption and anesthetic recovery times were reduced with depth of anesthesia monitoring [17]. Our findings of lower cost of propofol as induction agents when BIS monitoring was used, suggest that monitoring depth of anesthesia may lead to dose reduction during induction of anesthesia. The lower hourly cost of fentanyl during anesthesia in groups SEVO and SEVO+ compared to PROP and PROP+ may be explained by the analgesic effect of sevoflurane. The hourly cost of fentanyl, atracurium, as well as cost of reversal and postoperative analgesics did not appear to be influenced by the use of depth of anesthesia monitoring. This may be explained by similar patient (e.g. age, body weight) and surgical characteristics (type of surgery, time of surgery and anesthesia) in all groups. In addition, neuromuscular block reversal was administered to all patients. Sevoflurane anesthesia without BIS monitoring (group SEVO) was associated with the lowest cost, whereas propofol anesthesia with BIS monitoring (group PROP+) was the most expensive. The reason for this is the higher costs of the intravenous technique and of the disposable BIS sensor. In contrast to other studies, in our study the incidence of perioperative minor complication was similar in all groups [16, 17]. These differences with literature may be explained to the small number of recruited patients.

Karaca et al. found that time to spontaneous breathing was shorter and time to eye opening was longer when BIS was used compared to the control group, although these differences were not statistically significant [18]. They concluded that it was difficult to say whether there was a significant difference in terms of total cost when BIS monitoring was used. In their opinion, the most important factor in BIS choice is the indication (e.g. awake craniotomy surgeries, patients with a previous history of intraoperative awareness, or surgeries having a high risk of awareness). In these cases, BIS should be used without considering the cost. Our results showed that time to extubation were shorter when BIS and TOF monitoring was employed. Time to MAP restoration was independent of BIS monitoring. Our results suggest that anesthesia guided by BIS and TOF may provide faster postoperative recovery.

The present prospective study has some limitations. First, many patients were excluded (not meeting inclusion criteria of study, e.g. type of surgery, age, ASA physical status, medical history) which may limit the interpretation of our results. Results are drawn from a relatively small number of patients and are restricted to anesthesia for ENT surgery. Other expenses, such as labor/salary for our department were not calculated. Therefore, conclusions related to the secondary outcomes should be interpreted with reservation. Cost of anesthesia was estimated based on the pharmacy prices in our hospital which may vary from other hospitals and other countries.

## Conclusions

In summary, our results suggest that intraoperative BIS and TOF monitoring may hasten postoperative recovery after ENT surgery. In our circumstance using BIS during general anesthesia was associated with lower drug use and higher disposable costs. Detailed cost analysis showed that BIS monitoring increased total cost of anesthesia. In certain patient populations, however, the advantage of intraoperative BIS monitoring may outweigh its cost. Further investigation is needed to clarify the benefits of BIS monitoring particularly in high-risk population such as patients with ASA III-IV and/or history of intraoperative awareness or increased hemodynamic risk. In addition, monitoring of depth of anesthesia may be more essential in ENT surgeries with controlled hypotension.

## Abbreviations

ASA: American Society of Anesthesiologists
BIS: Bispectral index
ECG: Electrocardiography
ENT: Ear-nose-throat
FGF: Fresh gas flow
FiO_2_: Inspired oxygen fraction
IV: Intravenously
MAC: Minimal alveolar concentration
MAP: Mean arterial blood pressure
PONV: Postoperative nausea and vomiting
PROP: Propofol anesthesia
PROP+: Propofol anesthesia with additional monitoring
SD: Standard deviation
SEVO: Sevoflurane anesthesia
SEVO+: Sevoflurane anesthesia with additional monitoring
SpO_2_: Peripheral capillary oxygen saturation
TOF: Train-of-four
